# Association between nighttime sleep duration trajectories and frailty in middle-aged and older adults: A work-in-progress model based on a CHARLS cohort

**DOI:** 10.1371/journal.pone.0339843

**Published:** 2025-12-30

**Authors:** Yanling Zhou, Xiucheng Guo, Sunjuan Dong, Yimeng Wang, Mingming Zheng, Chi Wang, Li Wu, Weiting Liu

**Affiliations:** 1 School of Nursing, Anhui University of Chinese Medicine, Hefei, China; 2 Department of Orthopedics, The Second Affiliated Hospital of Shandong First Medical University, Taian, China; 3 School of Psychology, Northwest Normal University, Lanzhou, China; 4 Department of Sleep Disorders, Affiliated Psychological Hospital of Anhui Medical University, Hefei, China; 5 Hefei Fourth People’s Hospital, Hefei, China; 6 Key Laboratory of Geriatric Nursing and Health, Anhui University of Chinese Medicine, Hefei, China; UNAM Facultad de Estudios Superiores Zaragoza: Universidad Nacional Autonoma de Mexico Facultad de Estudios Superiores Zaragoza, MEXICO

## Abstract

**Background:**

Frailty is a major public health issue that impairs the quality of life and health outcomes of middle-aged and older adults. Changes in sleep patterns may play a critical role in the development of frailty. However, studies on the relationship between nighttime sleep duration trajectories and frailty risk remain limited. This study aimed to explore this association and provide preliminary evidence for frailty prevention and intervention.

**Methods:**

Data were obtained from 8083 participants aged ≥ 45 years in the China Health and Retirement Longitudinal Study (CHARLS), assessed at four time points over seven years. Latent class trajectory modeling was applied to identify distinct nighttime sleep duration trajectories. Frailty was measured using a frailty index comprising 32 health deficits. Logistic regression was used to evaluate the associations between sleep trajectories and frailty.

**Results:**

This study identified two nighttime sleep duration trajectories: normal stable trajectory group, encompassing 7137 (88.2%) individuals, and short with gradual increasing trajectory group, including 946 (11.7%) individuals. Compared with normal stable trajectory group, participants in short with gradual increasing trajectory group had a significantly higher incidence of frailty. Furthermore, after adjusting for covariates, this association remained statistically significant, although the effect size was modest.

**Conclusions:**

Long-term exposure to short sleep may be associated with an elevated risk factor for frailty in middle-aged and older adults. Given the modest effect sizes observed, these findings should be interpreted as exploratory and part of a work-in-progress model. Continuous monitoring of sleep duration may still offer insights for early screening and prevention of frailty.

## 1. Introduction

With the accelerating global trend of population aging, China is also facing a severe aging challenge and is among the countries most affected by this demographic shift. According to the latest data from the National Bureau of Statistics, by the end of 2024, the population aged 60 and above in China had reached 300 million, accounting for 22.0% of the total population, while the proportion of those aged 65 and over rose to 15.6% [1]. This demographic shift poses significant challenges to the country’s pension and healthcare systems, including heavier burdens of chronic disease management, rising demand for medical services, insufficient long-term care infrastructure, and increasing pressure on healthcare workforce allocation. In response to these challenges, the “Healthy China 2030” blueprint has, for the first time, incorporated the concept of “healthy aging” into the national strategic framework [2]. Healthy aging refers to the process by which older adults maintain physical and mental well-being and actively participate in social activities, thereby sustaining a dynamic balance between individual health and societal development [3]. This concept goes beyond the absence of major illness or physical decline, emphasizing multidimensional health across physical, psychological, cognitive, and social domains. Its core lies in mitigating age-related health deterioration through proactive risk prevention and management, thereby preventing adverse health outcomes such as frailty and disability, while maintaining quality of life and independence among older adults [4]. Extensive research has shown that frailty significantly increases the risk of disability, all-cause mortality, hospitalizations, and long-term care dependence [5]. Consequently, frailty has emerged as one of the greatest challenges in achieving healthy aging and represents a major public health concern for middle-aged and older adults [6].

Frailty is a critical age-related clinical syndrome characterized by diminished physiological reserves across multiple systems, increased physical vulnerability, impaired ability to maintain homeostasis, and heightened susceptibility to stressors and adverse clinical outcomes [7]. Frailty is typically diagnosed through multidimensional assessments. Currently, two diagnostic approaches are most widely used in clinical practice: (1) the Fried phenotype model, proposed by Fried et al., which identifies frailty based on five key criteria—unintentional weight loss, self-reported exhaustion, decreased grip strength, slow walking speed, and low physical activity. Individuals meeting three or more of these criteria are classified as frail [8]; (2) the Frailty Index (FI), developed by Rockwood, which is grounded in the deficit accumulation model. The FI quantifies frailty by assessing the cumulative number of health deficits, including symptoms, diseases, and functional impairments, to reflect an individual’s overall vulnerability to adverse outcomes [9]. Compared with the Fried phenotype, the FI offers greater predictive power for adverse health outcomes, as it provides a more comprehensive assessment of frailty severity, incorporates comorbidities and disabilities, and allows for finer gradations of risk [10]. The prevalence of frailty increases with age, and epidemiological studies have shown that the overall prevalence can be as high as 40% [11]. Moreover, frailty is closely associated with adverse health outcomes such as falls, disability, and mortality, and it significantly contributes to the increasing burden on healthcare systems and social services [12,13]. Therefore, it is an urgent public health task to actively address the problem of frailty. Distinct from the natural aging process, frailty exhibits features of reversibility and preventability. It can be alleviated or even reversed through timely identification and appropriate intervention. Thus, identifying modifiable risk factors for frailty is essential for developing effective prevention and intervention strategies. In addition to demographic characteristics associated with frailty, lifestyle factors may also contribute to its development [14], among which sleep duration has emerged as a potentially important factor affecting frailty [15].

Sleep constitutes a fundamental physiological requirement for human homeostasis, playing a crucial role in regulating and maintaining systemic equilibrium. Particularly for middle-aged and older adults, sleep duration is directly associated with physical and mental well-being. However, aging populations typically undergo characteristic alterations in sleep architecture, often manifesting as reduced nighttime sleep duration and increased frequency of nighttime awakenings [16,17]. A recent meta-analysis of Chinese populations revealed that 45.5% of adults experience short sleep duration (< 6 hours), while 17.6% demonstrate prolonged sleep patterns (> 9 hours) [18], indicating substantial variability in sleep patterns. Currently, several studies have shown that both short and long sleep durations not only affect the quality of daily life but may also significantly impair physiological functions and increase the risk of frailty [19–21]. Therefore, sleep-related issues are increasingly recognized as critical factors that threaten human health. In recent years, many researchers have increasingly focused on the temporal relationship between sleep duration and frailty progression. Several studies have reported a potential association between sleep duration and the development of frailty, although the findings remain inconsistent. For example, a large Japanese cross-sectional study found that both short sleep duration (≤ 6 hours) and long sleep duration (≥ 9 hours) were associated with increased frailty risk [22], a finding echoed by a cohort study from the Netherlands [23]. In contrast, a study based on the National Health and Nutrition Examination Survey (NHANES) in the United States indicated that only sleep durations of ≥ 10 hours were significantly associated with increased frailty risk [24]. Similarly, a multicenter cohort study in Korea reported that sleep durations longer than 8 hours were independently associated with an increased frailty risk [25]. These discrepancies may be attributed to differences in the demographic characteristics of study populations, the length of follow-up, and the covariates adjusted in the analyses.

Despite the growing body of research, several limitations remain in the literature. First, most previous studies have adopted a cross-sectional design and relied on sleep duration data from a single time point, failing to capture the dynamic nature of sleep over time and its potential impact on frailty. Single-time-point measurements may not accurately capture the long-term effects of sleep on frailty [26]. Second, while some longitudinal studies have explored the association between sleep duration and frailty, they often focus on population-level average changes in sleep duration, neglecting the heterogeneity within the population [27]. Third, existing research has shown that different sleep trajectory patterns have varying effects on hypertension, diabetes, cardiovascular events, and all-cause mortality [28]. Thus, considering the diversity of sleep trajectories and individual differences is essential when examining the relationship between sleep duration and frailty. In recent years, some studies have begun to focus on the dynamic changes in sleep duration, highlighting the importance of long-term sleep patterns in understanding the complex relationship between sleep duration and health outcomes [29,30]. Latent class trajectory modeling (LCTM) is a commonly used method for describing trajectories of exposure over the life course. It enables the identification of distinct patterns of change within a population over time while accounting for heterogeneity among individuals [31,32].

This study aims to apply LCTM to analyze the trajectoryof nighttime sleep duration among middle-aged and elderly adults and to investigate how these sleep trajectories are associated with frailty, in order to provide evidence for early identification and prevention of frailty in aging populations.

## 2. Materials and methods

### 2.1 Study population

**Fig 1:**
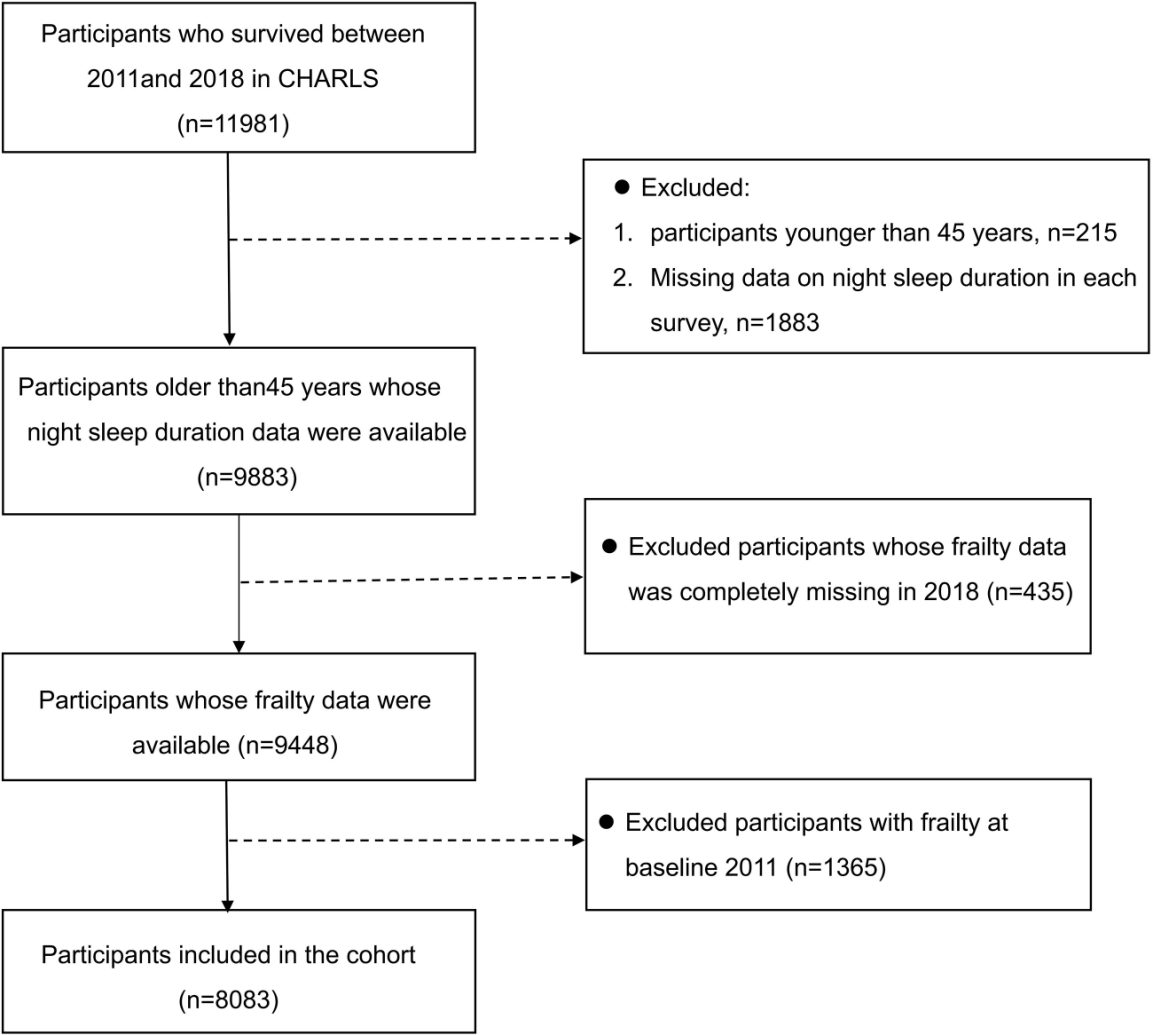
Flow chart for inclusion and exclusion of research subjects.

We used the data from the China Health and Retirement Longitudinal Study (CHARLS) in 2011, 2013, 2015, and 2018. CHARLS is a nationally representative longitudinal survey targeting Chinese community-dwelling residents aged 45 years and over. It collects comprehensive information on demographics, socioeconomic status, behavioral factors, health-related indicators, and biomarkers. The baseline survey in 2011, administered by the National School of Development at Peking University, recruited 17,708 participants from 10,257 households across 450 villages within 150 county-level units spanning 28 provinces. The study achieved an 80.5% response rate. Follow-up surveys have been conducted every 2–3 years, and the data are made publicly available one year after each wave is completed (http://charls.pku.edu.cn/). The questionnaire design was informed by international standards, and both the response rate and data quality are among the highest worldwide for comparable surveys. CHARLS data have been widely used and recognized in the academic community [33]. All study procedures were adhered to the Strengthening the Reporting of Observational Studies in Epidemiology (STROBE) guidelines [34]. In our study, we included middle-aged and older adults aged 45 years and above as the study population, resulting in a final sample of 8,083 participants who completed all four survey waves and met the inclusion criteria, and the detailed selection flowchart is shown in Fig 1.

### 2.2 Ethics approval and consent to participate

CHARLS has been approved by the Ethics Committee of Peking University (No.IRB00001052–11015). All participants signed a written informed consent form at the time of participation, and their information was kept anonymous.

### 2.3 Assessment of nighttime sleep duration

Nighttime sleep duration was assessed through standardized CHARLS questionnaires by querying: “During the past month, how many hours of actual sleep did you get on average per night?” Self-reported nighttime sleep duration has been widely used in previous studies and is considered to have acceptable reliability [35]. Prior research has demonstrated a moderate correlation between self-reported sleep duration and objective measures, such as actigraphy-based assessments, supporting its use as a practical and feasible tool in epidemiological studies [36,37]. Based on data collected in 2011, 2013, 2015, and 2018, we employed LCTM to identify patterns in the trajectories of nighttime sleep duration over time [38].

### 2.4 Frailty index

In this study, we used FI to assess frailty, which is based on the cumulative deficit model of aging. The FI quantifies frailty by calculating the proportion of health deficits an individual has accumulated. While the specific items used to construct the FI can be flexibly selected according to the principles for identifying health deficits, a valid FI should include at least 30 health-related variables [39]. The FI has been used in several CHARLS studies [40,41]. Following established FI construction criteria [42], we selected 32 multidimensional indicators encompassing physical functioning, cognitive-psychological status, and comorbid conditions (detailed variables and coding provided in S1 Table). The FI was computed by summing all deficit scores and dividing by the total number of items, producing values ranging from 0 (robust) to 1 (most frail). Consistent with previous epidemiological thresholds [43], frailty was operationalized using FI > 0.25.

### 2.5 Covariates

The covariates in this study included sociodemographic characteristics, health behaviors, and health-related factors. Specifically, sociodemographic characteristics included age(continuous), sex (male/female), body mass index (BMI, kg/m²), marital status (married/unmarried), place of residence (rural/urban), living arrangement (living alone/living with others), educational level (illiterate/primary school or below/junior high school/senior high school or above), and annual per capita household expenditure level (tertiles). Health behaviors and health-related factors include current smoking status, current alcohol consumption, participation in physical activities, number of social activities engaged in, and presence of chronic pain. All covariates were obtained from the CHARLS face-to-face questionnaire or physical examination. Chronic pain was assessed with the question, “Are you often troubled by physical pain?” (responses: yes/no); BMI was calculated from measured height and weight; physical activity and social activity were assessed based on questionnaire items. Detailed procedures and measurement methods are described in the CHARLS study protocol [44]. Missing covariate data were handled through fully conditional specification using multiple imputation by chained equations (MICE) with 50 iterations and 10 imputed datasets, incorporating auxiliary variables to satisfy the missing-at-random assumption.

### 2.6 Statistical analyses

We employed LCTM to identify distinct subgroups exhibiting similar trajectories in nighttime sleep duration changes. The number of trajectory classes was determined by comparing model fit indices, including the Bayesian Information Criterion (BIC), Akaike Information Criterion (AIC), aBIC (adjusted Bayesian Information Criterion) and entropy. Lower BIC, aBIC and AIC values indicate better model fit, while entropy ranges from 0 to 1, with values closer to 1 indicating clearer classification and higher accuracy in assigning individuals to different trajectory classes [45]. In addition, each trajectory group was required to comprise more than 5% of the total sample size to be considered acceptable. Based on a comprehensive evaluation of these model fit indices, the optimal trajectory model and classification were selected.

The basic characteristics among participants with different sleep duration trajectories were compared using one-way ANOVA or the Kruskal-Wallis test for continuous variables and the Chi-square test for categorical variables. Logistic regression models were used to examine the associations between sleep trajectory groups and frailty, and the odds ratio (OR) and 95% confidence interval (CI) were computed. Model 1 was adjusted for demographic variables, including age, sex, residence, marital status, living arrangement, and education level. Model 2 was further adjusted for health behaviors and socioeconomic factors (e.g., BMI, smoking, alcohol consumption, physical activity, household expenditure, and social engagement). Model 3 additionally adjusted for health-related factors (e.g., chronic pain). Next, We performed subgroup analyses to explore whether the association between nighttime sleep duration trajectories and frailty differed by age, gender, smoking behavior, drinking behavior, BMI, and chronic pain. In addition, the *p-*value of interaction effect was calculated using likelihood ratio test to explore the interaction between nighttime sleep duration trajectory and age, gender, smoking behavior, drinking behavior, BMI and chronic pain.

The LCTM was constructed using the “lcmm” package in the R (version 4.2.2) software [46]. Statistical analyses were performed using STATA 18.0和 R 4.2.2, and all tests were two-sided with *p-*value < 0.05 indicating statistical significance.

## 3. Results

### 3.1 Nighttime sleep duration trajectories

**Fig 2:**
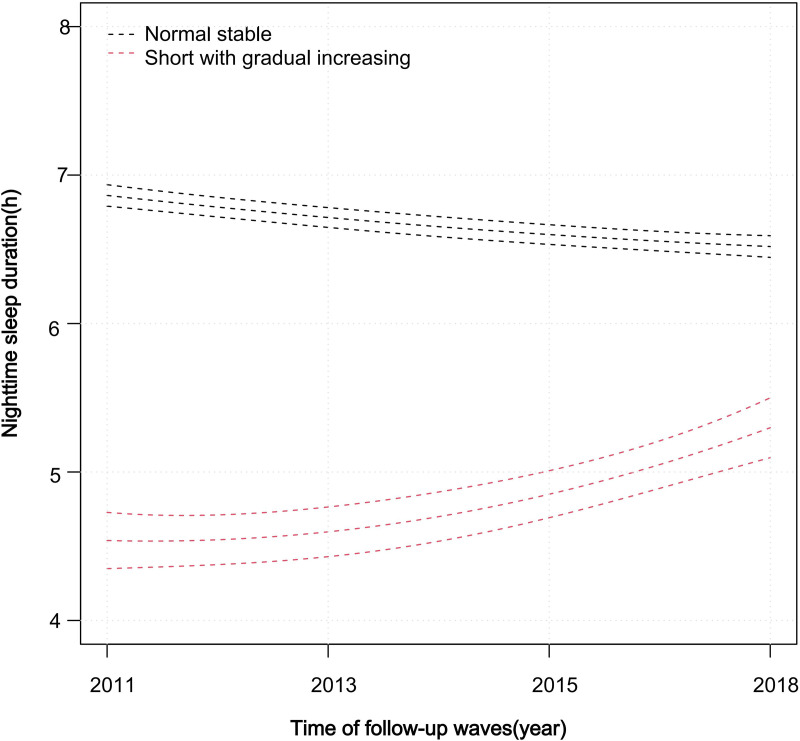
Nighttime sleep duration trajectories from 2011 to 2018 in the Chinese middle-aged and older adults. Notes: Two trajectory groups: the normal stable trajectory group (88.2%, n = 7,137); the short with gradual increasing trajectory group (11.7%, n = 946). The lines in the figure represent the temporal trends in nighttime sleep duration for each trajectory group.

To determine the optimal trajectory model for nighttime sleep duration, we used LCTM to fit and compare latent class trajectory models with 1–3 classes (fit indices are shown in S2 Table). Although the 3-class model had a higher entropy than the 2-class model, and AIC, BIC, and aBIC values continued to decrease, one of its trajectory groups had a very small sample size (1.57%, approximately 127 participants). Considering model interpretability and practical significance, we ultimately selected the two-class trajectory model for subsequent analyses. Fig 2 depicts the trajectories of nighttime sleep duration in detail. Based on the initial levels and developmental trends of each trajectory, they were labeled as: normal stable trajectory group (88.2%, n = 7,137) and short with gradual increasing trajectory group (11.7%, n = 946). To examine the specific changes in sleep duration across trajectory groups, we calculated the baseline and endpoint estimates as well as the within-group changes (see S3 Table). Specifically, the average nighttime sleep duration in the short with gradual increasing trajectory group increased from 4.8 ± 0.9 hours in 2011 to 6.1 ± 0.8 hours in 2018, with a mean change of 1.11 hours (95% CI: 0.97–1.26). In contrast, the normal stable trajectory group maintained a relatively constant sleep duration throughout the follow-up, ranging from 6.9 ± 1.5 hours in 2011 to 6.5 ± 1.8 hours in 2018, with a minimal mean change of 0.41 hours (95% CI: −0.46 to −0.37). These results provide a clear depiction of the magnitude of sleep changes over time for the two trajectory groups.

### 3.2 Baseline characteristics of trajectory subpopulation

The baseline characteristics of participants according to nighttime sleep duration trajectories are shown in Table 1. Notably, Compared to those in the normal stable trajectory group, individuals in the short with gradual increasing trajectory group were more likely to be female, older, unmarried, have lower educational attainment, lower per-capita household expenditure, and not engaging in smoking, alcohol consumption, or social activities. In addition, participants in this trajectory exhibited a relatively lower BMI and were more likely to report chronic pain (*p* < 0.05).

**Table 1:** Baseline characteristics of participants based on nighttime sleep duration trajectories.

Variables	Overall n = 8083	Normal stable trajectory group n = 7137	Short with gradual increasing trajectory group n = 946	*p-*value^a^
Age, M (P25, P75)	57 (50, 63)	57 (50, 63)	59 (53, 65)	< 0.001
Sex, n (%)				< 0.001
Male	3950 (48.87)	3579 (50.15)	371 (39.22)	
Female	4133 (51.13)	3558 (49.85)	575 (60.78)	
Marital status, n (%)				<0.001
Others	690 (8.540)	574 (8.04)	116 (12.26)	
Married	7393 (91.46)	6563 (91.96)	830 (87.74)	
Residence status, n (%)				0.115
Urban	2921 (36.15)	2601 (36.44)	320 (33.83)	
Rural	5162 (63.85)	4536 (63.56)	626 (66.17)	
Co-residence, n (%)				0.129
Alone	3006 (37.19)	2633 (36.89)	373 (39.43)	
With others	5077 (62.81)	4504 (63.11)	573 (60.57)	
Education level, n (%)				< 0.001
Illiterate	1846 (22.84)	1639 (22.96)	207 (21.88)	
Elementary school or below	3361 (41.58)	2826 (39.6)	535 (56.55)	
Middle school or below	1882 (23.29)	1732 (24.27)	150 (15.86)	
High school or above	994 (12.29)	940 (13.17)	54 (5.71)	
Per capita household expenditure level M (P25, P75)	5750 (3100, 7600)	5850 (3150, 7705)	5060 (2772, 7050)	< 0.001
Smoke at present, n (%)				
Yes	3206 (39.66)	2892 (40.52)	314 (33.19)	
No	4877 (60.34)	4245 (59.48)	632 (66.81)	< 0.001
Drink at present, n (%)				< 0.001
Yes	2850 (35.25)	2565 (35.94)	285 (30.13)	
No	5233 (64.75)	4572 (64.06)	661 (69.87)	
Exercise at present, n (%)				0.121
Yes	7432 (91.94)	6550 (91.78)	882 (93.23)	
No	651 (8.06)	587 (8.22)	64 (6.77)	
Chronic pain, n (%)				< 0.001
Yes	2182 (26.99)	1786 (25.02)	396 (41.86)	
No	5901 (73.01)	5351 (74.98)	550 (58.14)	
Number of social activities, M (P25, P75)	1 (0, 1)	1(0, 1)	0 (0, 1)	0.002
BMI M (P25, P75)	23.77 (21.31, 25.41)	23.834 (21.379, 25.45)	23.295 (20.776, 24.869)	< 0.001

^a^Based on a Wilcoxon signed-rank test or Chi-square statistics as appropriate.

### 3.3 Association of nighttime sleep duration trajectories with frailty

**Fig 3:**
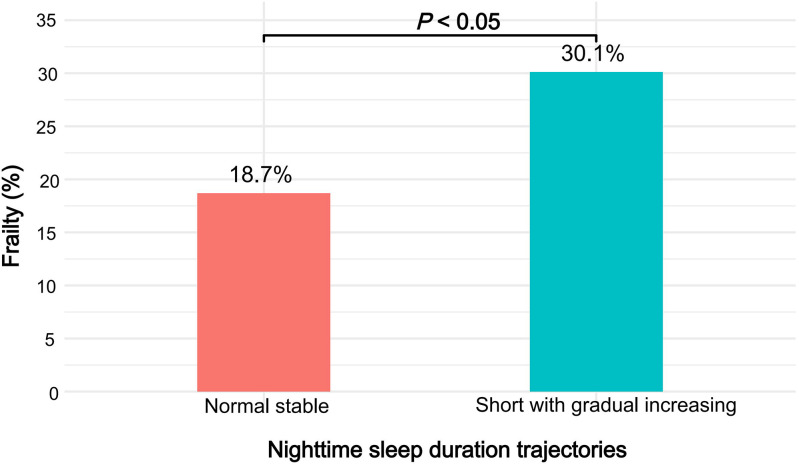
Frailty rates according to nighttime sleep duration trajectories. Notes: This figure illustrates the incidence of frailty across the two identified nighttime sleep duration trajectory groups over four survey waves (2011, 2013, 2015, and 2018) among Chinese middle-aged and older adults.

Table 2 presents the associations between nighttime sleep duration trajectories and frailty. The incidence of frailty was 18.7% in the normal stable trajectory group and 30.1% in the short with gradual increasing trajectory group (Fig 3). After adjusting for sociodemographic characteristics (Model 1), the OR for frailty in the normal stable group was 0.648(95% CI: 0.553–0.758). Upon further adjustment for lifestyle and socioeconomic factors (Model 2), the association between nighttime sleep duration and frailty remained statistically significant; the OR for frailty in the normal stable trajectory group was 0.651 (95% CI: 0.556–0.762). Furthermore, even after additional adjustment for health-related factors (Model 3), nighttime sleep duration continued to exhibit a significant association with frailty; the OR for frailty in the normal stable trajectory group was 0.726 (95% CI: 0.618–0.853). Additionally, in the normal stable trajectory group, nighttime sleep duration remained relatively stable throughout, potentially contributing to their lower frailty risk.

**Table 2:** Logistic regression models for frailty and nighttime sleep duration trajectories.

	Normal stable trajectory group	Short with gradual increasing trajectory group
Subjects, n	7137	946
Frailty cases, n	1335	285
OR (95% CI)		
Model 1^a^	0.648 (0.553-0.758)	Reference
Model 2^b^	0.651 (0.556-0.762)	Reference
Model 3^c^	0.726 (0.618-0.853)	Reference

^a^Model 1 was adjusted for participants’ demographic variables, including age, sex, residence, marital status, living arrangement, and educational level; ^b^ Model 2 was1 was additionally adjusted for health behaviors and socioeconomic factors (such as BMI, smoking, drinking, physical activity, household expenditure, and social engagement); ^c^ Model 3 was additionally adjusted for health-related factors (chronic pain); OR: odds ratio; CI: confidence interval.

### 3.4 Subgroup analysis

**Fig 4:**
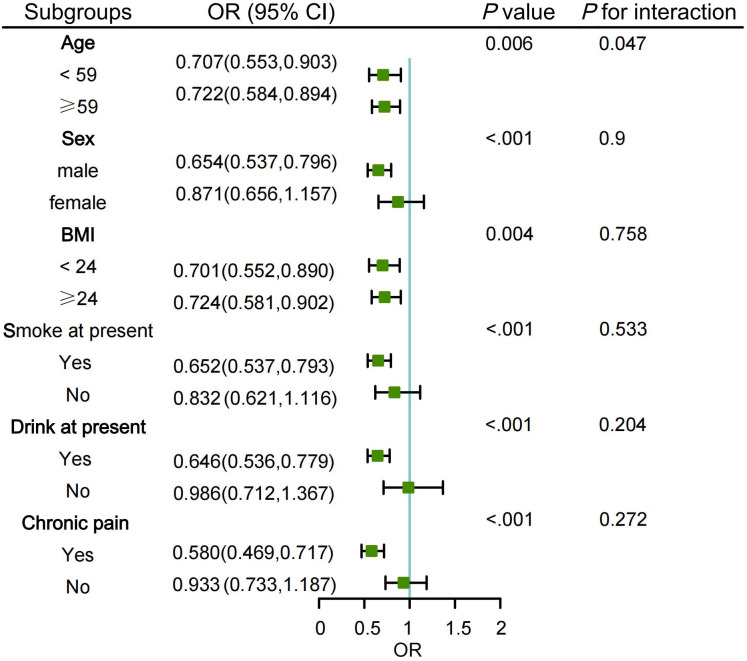
Subgroup analysis of the association between nighttime sleep duration trajectories and frailty risk. Notes: The Normal stable trajectory group was used as the reference. The model was adjusted for age, sex, BMI, smoking status, alcohol consumption, and chronic pain.

Fig 4 displays the outcomes of subgroup analyses. These findings indicate a statistically significant relationship between short with gradual increase trajectories and frailty among specific subgroups including individuals who were male, currently smoked, currently consumed alcohol, and had chronic pain. Within these subgroups, the trajectory was significantly associated with increased frailty risk. Notably, nighttime sleep duration trajectories exhibited a weak interaction with age (*p* for interaction = 0.047).

### 3.5 Sensitivity analysis

After excluding participants aged over 80 years in the baseline survey, the trajectories of the two groups (S1 Fig) and the results of the correlation analyses (S4 Table) remained consistent with the original findings. The E-values of Model 1, Model 2, and Model 3 were 2.46 (confidence interval, 3.02), 2.44 (confidence interval, 2.99), and 2.10 (confidence interval, 2.62), respectively. These results suggest that only an unmeasured confounder associated with both the exposure and the outcome by a risk ratio greater than the respective E-value could fully explain away the observed associations, suggesting that the observed associations are robust to potential unmeasured confounders.

## 4. Discussion

In this study, we used LCTM to explore the correlation between nighttime sleep duration trajectories and frailty in middle-aged and older adults based on data from the CHARLS cohort from 2011 to 2018. Ultimately, the analysis identified two distinct trajectories of nighttime sleep duration, including a normal stable trajectory group and short with gradual increasing trajectory group. Our results revealed a higher frailty incidence in the short with gradual increasing trajectory group (30.1%) compared to the normal stable trajectory group (18.7%). Even after progressively adjusting for demographic characteristics, lifestyle factors, and health-related variables, this association remained significant. Although the difference in frailty incidence between the two groups was slightly over 11%, given the large middle-aged and older population in China, this could correspond to millions of older adults at elevated risk of frailty. Moreover, long-term short sleep can accumulate adverse effects through pathways such as chronic inflammation, sarcopenia, and neuroendocrine imbalance [47,48]. Even if the difference is modest, it may nonetheless impose a substantial health burden. Therefore, early identification of high-risk individuals and the implementation of sleep interventions remain important for public health.

Stable and moderate sleep serves as the foundation for maintaining cognitive function and the normal operation of various bodily systems, thereby helping to delay functional decline. In contrast, long-term short sleep leads to the accumulation of sleep debt, adversely affecting multiple physiological systems and thereby increasing the risk of frailty [49]. Our findings are consistent with previous studies [50,51]. Ensrud and colleagues conducted a cross-sectional survey of 3,133 participants and found that persistently short sleep duration (≤ 5 hours) increased the risk of frailty [52]. Another longitudinal cohort study based on CHARLS examined the relationship between sleep duration and frailty among middle-aged and older Chinese adults, demonstrating that short sleep duration was associated with increased frailty risk in this population [27]. By employing latent class trajectory analysis, the present study provides a more comprehensive capture of the dynamic changes in sleep patterns and their health effects, emphasizing the importance of regular sleep for healthy aging and providing key evidence for frailty prevention strategies in middle-aged and older adults.

Given that sleep is a cumulative and long-term physiological process, it is essential to investigate the impact of sustained changes in nighttime sleep duration on frailty. Our findings suggest that individuals in the normal stable trajectory group had a lower risk of developing frailty compared to those in the short with gradually increasing trajectory group. Long-term short sleep may promote the development of frailty through multiple biological pathways. First, inflammatory responses are considered a key mechanism [53]. Studies have shown that persistent short sleep can induce low-grade systemic inflammation, increasing levels of C-reactive protein, IL-6, and TNF-α in the blood, thereby exacerbating chronic systemic inflammation, particularly in older and frail individuals, and promoting the onset of frailty [48,54]. Second, chronic sleep insufficiency can impair muscle mass and function, increasing the risk of sarcopenia, a core component of frailty [55]. Sleep deprivation can activate the hypothalamic-pituitary-adrenal axis, leading to sustained elevation of glucocorticoids, which accelerates muscle atrophy and mitochondrial dysfunction, while also suppressing hypothalamic-pituitary-gonadal axis activity, reducing growth hormone and testosterone secretion, further impairing muscle protein synthesis and reducing bone mass [56,57]. Additionally, insufficient sleep may reduce natural killer cell activity, increase insulin resistance, and cause lipid metabolism disorders, affecting energy reserves and healthy aging [58]. It can also impair cognitive function, increasing the risk of falls and decline in activities of daily living, thereby further accelerating frailty progression [59]. Conversely, frailty itself may also affect sleep. Frail individuals often experience sleep interruptions or insufficiency due to chronic pain, nocturnal awakenings, and chronic disease burden, forming a vicious cycle between sleep disturbance and frailty [28]. Notably, in this study, although the short sleep gradually increasing trajectory group showed some extension of nighttime sleep duration in later stages, their risk of frailty remained significantly elevated. This may be because chronic sleep deprivation in early stages already disrupts intracellular neuronal metabolism and cognitive function, and compensatory sleep may be insufficient to fully reverse the damage [60]. Moreover, longer sleep duration does not necessarily indicate better sleep quality. In some individuals, increased sleep time with aging may reflect prodromal symptoms of somatic diseases or metabolic disturbances, potentially further increasing frailty risk [61]. Therefore, early identification of sleep disturbances and timely intervention are essential for preventing the onset of frailty.

The association between sleep and frailty is influenced not only by individual physiological and psychological factors but also by cultural factors. First, in today’s fast-paced lifestyle, nighttime sleep duration has generally declined [62]. Older adults, in particular, experience more pronounced sleep problems due to health issues, medication effects, and life transitions such as retirement, bereavement, and reduced social participation [63]. Second, many older adults bear responsibilities such as caring for grandchildren, which can enhance their sense of social value but may also emotional burdens and impose chronic stress, indirectly affecting physical function [64]. At the same time, under the influence of collectivist culture, older adults often prioritize family interests over personal health, potentially leading them neglect their own health and delay interventions, further accelerating the progression of frailty [65]. Therefore, future interventions targeting frailty in older adults should fully consider cultural influences. For example, sleep health education could be integrated with the traditional Chinese theory, community activity platforms could be established to encourage older adults to proactively express health concerns, and the “preventive treatment of disease” concept could be applied to implement sleep interventions in the early stages of frailty, thereby preventing or delaying its development.

Furthermore, subgroup analyses indicated that in the subgroups of younger age, male sex, current smokers, current drinkers, and individuals with chronic pain, the short with gradual increasing trajectory group was significantly associated with increased frailty risk. Notably, among participants younger than 59 years, the association between the short with gradual increasing trajectory group and frailty was more pronounced (OR = 0.707), suggesting that middle-aged adults may be more sensitive to changes in sleep duration. On one hand, the physical function of younger adults has not yet declined significantly, and they may overlook changes in their sleep patterns; prolonged sleep insufficiency could accelerate depletion of physiological reserves, leading to earlier onset of frailty symptoms. At the same time, only the age subgroup showed a statistically significant interaction (*p for* interaction = 0.047), indicating that age may play a moderating role in the association between sleep trajectories and frailty. This is consistent with the “frailty index” theory, which posits that the accumulation of health deficits can accelerate exponentially even over a decade within the same timeframe [9]. Moreover, studies have shown that in cohorts aged ≥ 45 years, age heterogeneity within the sample can weaken the statistical significance of age itself, although its biological relevance remains [66], supporting the biological rationale for retaining age as a variable in the model.

This study has several strengths. While dynamic changes in sleep duration over time may carry more clinical relevance than sleep status measured at a single time point, traditional longitudinal studies have typically focused on average changes at the population level. In contrast, our study analyzed repeated measures of nighttime sleep duration and employed latent class trajectory modeling to identify subgroups of individuals with similar temporal sleep patterns. This approach not only allows characterization of heterogeneous sleep trajectories but also provides a nuanced understanding of how varying sleep patterns influence health outcomes differently across subpopulations. However, several limitations should be acknowledged. First, nighttime sleep duration was self-reported and may be subject to recall bias or subjective misperception, potentially introducing measurement error. Future research could incorporate objective assessments such as polysomnography or wearable sleep trackers to enhance data accuracy. Second, this study focused solely on the nighttime sleep duration, while sleep quality, an important confounding factor, was not considered due to data limitations. Future studies should examine the effects of sleep quality or sleep pattern trajectories on frailty using validated instruments such as the pittsburgh sleep quality index, sleep diaries, or device-based monitoring. Third, our study included only middle-aged and older Chinese adults, which may limit the generalizability of the findings to other age groups or ethnic populations. Further research is needed to examine whether the observed associations hold true across diverse demographic contexts. In addition, given that the observed numerical differences and effect sizes were relatively modest, the present findings should be interpreted cautiously. The model can be considered preliminary, and further validation in independent cohorts is warranted.

Despite the limitations mentioned above, this study holds significant clinical and public health implications. Frailty is a critical syndrome affecting the quality of life and health outcomes in middle-aged and older adults, and early identification and intervention can help delay its onset and progression, reducing healthcare burdens and social costs. Our findings suggest that trajectories of nighttime sleep duration could serve as early predictive indicators of frailty, and we recommend incorporating them into the core indicator system of health assessments for middle-aged and older adults to allow comprehensive and dynamic evaluation of frailty risk. In addition, community-based “sleep–frailty” integrated clinics could be established to provide chronic disease management and sleep health education, offering personalized interventions for high-risk older adults. Furthermore, leveraging the powerful data analysis capabilities of artificial intelligence, long-term sleep data could be recorded via wearable devices or mobile apps, enabling analysis of sleep pattern trends, prediction of sleep disorder risk, and facilitating early identification and intervention in frailty.

## 5. Conclusion

Our study found that, compared with the stable moderate sleep duration trajectory group, participants in the short sleep duration with gradual extension trajectory group had a modestly increased risk of frailty. This finding suggests a potential association between persistently short sleep duration and frailty in middle-aged and older adults, although the effect size was relatively small and should be interpreted with caution. Therefore, in future clinical practice, attention should be given to the dynamic changes in sleep duration among the middle-aged and elderly population, and sleep monitoring may be considered as a supplementary component of frailty risk assessment rather than a definitive screening tool at this stage. Individualized intervention plans, integrating factors such as nutrition and physical activity, may help support frailty prevention and healthy aging, but further research is needed to confirm these possibilities.

## Supporting information

S1 Fig Nighttime sleep duration trajectories from 2011 to 2018 in the Chinese middle-aged and older adults after excluding persons older than 80 years in the baseline survey. (TIFF)

S1 Table Items constituting the Frailty Index and cut-off points. (DOCX)

S2 Table Fit indices of latent class mixed model on nighttime sleep duration trajectories. (DOCX)

S3 Table Changes in nighttime sleep duration across trajectory groups from 2011 to 2018. (DOCX)

S4 Table Logistic regression models for frailty and nighttime sleep duration trajectories after excluding persons older than 80 years in the baseline vsurvey. ^a^ Model 1 was adjusted for participants’ demographic variables, including age, sex, residence, marital status, living arrangement, and educational level; ^b^ Model 2 was1 was additionally adjusted for health behaviors and socioeconomic factors (such as BMI, smoking, drinking, physical activity, household expenditure, and social engagement); ^c^ Model 3 was additionally adjusted for health-related factors (chronic pain); OR: odds ratio; CI: confidence interval. (DOCX)

## Abbreviations

CHARLS: China Health and Retirement Longitudinal Study
LCTM: Latent class trajectory model
FI: Frailty Index
STROBE: Strengthening the Reporting of Observational Studies in Epidemiology
BMI: Body Mass Index
MICE: Multiple Imputation by Chained Equations
BIC: Bayesian Information Criterion
AIC: Akaike Information Criterion
aBIC: adjusted Bayesian Information Criterion
CI: Confidence Intervals
OR: Odds Ratios.

## References

[1] National Bureau of Statistics of China. 2024 National Economic Operation Press Conference [Internet]. 2025 Jan 17 [cited 2025 Feb 25]. Available from: http://www.stats.gov.cn

[2] Central Committee of the Communist Party of China, State Council. “Healthy China 2030” Planning Outline [Internet]. 2016 Oct 25 [cited 2025 May 7]. Available from: http://www.gov.cn/zhengce/2016-10/25/content_5124174.htm

[3] Rowe JW, Kahn RL. Human aging: usual and successful. Science. 1987;237(4811):143–9. doi: 10.1126/science.3299702 3299702

[4] Estebsari F, Dastoorpoor M, Khalifehkandi ZR, Nouri A, Mostafaei D, Hosseini M, et al. The Concept of Successful Aging: A Review Article. Curr Aging Sci. 2020;13(1):4–10. doi: 10.2174/1874609812666191023130117 31657693 PMC7403646

[5] Alhwoaimel NA, Alqahtani BA, Alshehri MM, Alhowimel AS, Alenazi AM. Coexisting frailty and depression associated with low physical activity and quality of life in Saudi community-dwelling older adults: a cross-sectional study. Front Public Health. 2025;13:1531101. doi: 10.3389/fpubh.2025.1531101 40487528 PMC12141023

[6] YuBY, HuXM, MatalaR, MoYH, LiuJL, JinJG, et al. Association between trajectories of systolic blood pressure and frailty outcome in middle-aged and older adults. J Nutr Health Aging. 2024;28(5):100202. doi: 10.1016/j.jnha.2024.100202 38460319

[7] Khan KT, Hemati K, Donovan AL. Geriatric Physiology and the Frailty Syndrome. Anesthesiol Clin. 2019;37(3):453–74. doi: 10.1016/j.anclin.2019.04.006 31337478

[8] Fried LP, Tangen CM, Walston J, Newman AB, Hirsch C, Gottdiener J, et al. Frailty in older adults: evidence for a phenotype. J Gerontol A Biol Sci Med Sci. 2001;56(3):M146-56. doi: 10.1093/gerona/56.3.m146 11253156

[9] Rockwood K, Mitnitski A. Frailty in relation to the accumulation of deficits. J Gerontol A Biol Sci Med Sci. 2007;62(7):722–7. doi: 10.1093/gerona/62.7.722 17634318

[10] Chen X, Mao G, Leng SX. Frailty syndrome: an overview. Clin Interv Aging. 2014;9:433–41. doi: 10.2147/CIA.S45300 24672230 PMC3964027

[11] Deng Z, Hu Y, Duan L, Buyang Z, Huang Q, Fu X, et al. Causality between sleep traits and the risk of frailty: a Mendelian randomization study. Front Public Health. 2024;12. doi: 10.3389/fpubh.2024.1381482 PMC11112029 38784581

[12] Nemoto Y, Sato S, Kitabatake Y, Nakamura M, Takeda N, Maruo K, et al. Bidirectional relationship between insomnia and frailty in older adults: A 2-year longitudinal study. Arch Gerontol Geriatr. 2021;97:104519. doi: 10.1016/j.archger.2021.104519 34564037

[13] Zhao C, Wang Y, Jia X, Fan J, Wang N, Yang Y, et al. Associations of Dietary Diversity Trajectories with Frailty among Chinese Older Adults: A Latent Class Trajectory Analysis Based on a CLHLS Cohort. Nutrients. 2024;16(10):1445. doi: 10.3390/nu16101445 38794683 PMC11124478

[14] Yang G, Cao X, Li X, Zhang J, Ma C, Zhang N, et al. Association of Unhealthy Lifestyle and Childhood Adversity With Acceleration of Aging Among UK Biobank Participants. JAMA Netw Open. 2022;5(9):e2230690. doi: 10.1001/jamanetworkopen.2022.30690 36066889 PMC9449787

[15] Muhammad T, Lee S, Pai M, Mandal B. Association between sleep quality, sleep duration, and physical frailty among adults aged 50 years and older in India. BMC Public Health. 2024;24(1):3120. doi: 10.1186/s12889-024-20606-6 39529114 PMC11556025

[16] Li X, Xue Q, Yi X, Liu J. The interaction of occupational stress, mental health, and cytokine levels on sleep in Xinjiang oil workers: A cross-sectional study. Front Psychiatry. 2022;13:924471. doi: 10.3389/fpsyt.2022.924471 36245869 PMC9554706

[17] Li J, Vitiello MV, Gooneratne NS. Sleep in Normal Aging. Sleep Med Clin. 2018;13(1):1–11. doi: 10.1016/j.jsmc.2017.09.001 29412976 PMC5841578

[18] Lu L, Wang S-B, Rao W-W, Ungvari GS, Ng CH, Chiu HFK, et al. Sleep Duration and Patterns in Chinese Older Adults: a Comprehensive Meta-analysis. Int J Biol Sci. 2017;13(6):682–9. doi: 10.7150/ijbs.19695 28655994 PMC5485624

[19] Sabia S, Dugravot A, Léger D, Ben Hassen C, Kivimaki M, Singh-Manoux A. Association of sleep duration at age 50, 60, and 70 years with risk of multimorbidity in the UK: 25-year follow-up of the Whitehall II cohort study. PLoS Med. 2022;19(10):e1004109. doi: 10.1371/journal.pmed.1004109 36256607 PMC9578599

[20] Xie J, Li Y, Zhang Y, Vgontzas AN, Basta M, Chen B, et al. Sleep duration and metabolic syndrome: An updated systematic review and meta-analysis. Sleep Med Rev. 2021;59:101451. doi: 10.1016/j.smrv.2021.101451 33618187

[21] Zhao Y, Lu Y, Zhao W, Wang Y, Ge M, Zhou L, et al. Long sleep duration is associated with cognitive frailty among older community-dwelling adults: results from West China Health and Aging Trend study. BMC Geriatr. 2021;21(1):608. doi: 10.1186/s12877-021-02455-9 34706663 PMC8555015

[22] Nakakubo S, Makizako H, Doi T, Tsutsumimoto K, Hotta R, Lee S, et al. Long and Short Sleep Duration and Physical Frailty in Community-Dwelling Older Adults. J Nutr Health Aging. 2018;22(9):1066–71. doi: 10.1007/s12603-018-1116-3 30379304 PMC12275589

[23] van Oostrom SH, van der A DL, Rietman ML, Picavet HSJ, Lette M, Verschuren WMM, et al. A four-domain approach of frailty explored in the Doetinchem Cohort Study. BMC Geriatr. 2017;17(1):196. doi: 10.1186/s12877-017-0595-0 28854882 PMC5577839

[24] Baniak LM, Yang K, Choi J, Chasens ER. Long Sleep Duration Is Associated With Increased Frailty Risk in Older Community-Dwelling Adults. J Aging Health. 2020;32(1):42–51. doi: 10.1177/0898264318803470 30270714 PMC6440876

[25] Kang I, Kim S, Kim BS, Yoo J, Kim M, Won CW. Sleep Latency in Men and Sleep Duration in Women Can Be Frailty Markers in Community-Dwelling Older Adults: The Korean Frailty and Aging Cohort Study (KFACS). J Nutr Health Aging. 2019;23(1):63–7. doi: 10.1007/s12603-018-1109-2 30569070 PMC12280380

[26] de Souza ÂMN, Fernandes DP de S, Castro IS, Gróla FG, Ribeiro AQ. Sleep quality and duration and frailty in older adults: a systematic review. Front Public Health. 2025;13:1539849. doi: 10.3389/fpubh.2025.1539849 40078770 PMC11898741

[27] Huang L, He X, Zuo Y, Yang H, Zhang L. The relationship between sleep duration and frailty: findings from the China Health and Retirement Longitudinal Study. Front Public Health. 2024;12:1493533. doi: 10.3389/fpubh.2024.1493533 39764189 PMC11701061

[28] Wang Y-H, Wang J, Chen S-H, Li J-Q, Lu Q-D, Vitiello MV, et al. Association of Longitudinal Patterns of Habitual Sleep Duration With Risk of Cardiovascular Events and All-Cause Mortality. JAMA Netw Open. 2020;3(5):e205246. doi: 10.1001/jamanetworkopen.2020.5246 32442289 PMC7244989

[29] Baden MY, Hu FB, Vetter C, Schernhammer E, Redline S, Huang T. Sleep Duration Patterns in Early to Middle Adulthood and Subsequent Risk of Type 2 Diabetes in Women. Diabetes Care. 2020;43(6):1219–26. doi: 10.2337/dc19-2371 32209646 PMC7245349

[30] Wang Y, Hou W, Siddiqi SM, Sun C, Han T, Yang J. Association of sleep trajectory in adulthood with risk of hypertension and its related risk factors: the China Health and Nutrition Survey. J Clin Sleep Med. 2020;16(4):515–21. doi: 10.5664/jcsm.8254 32003742 PMC7161444

[31] Watson C, Geifman N, Renehan AG. Latent class trajectory modelling: impact of changes in model specification. Am J Transl Res. 2022;14(10):7593–606. 36398215 PMC9641469

[32] Wang W, Cheung S-H, Cheung SF, Sun RW, Hui CH, Ma HYD, et al. A systematic review and meta-analysis of group-based trajectory modeling of sleep duration across age groups and in relation to health outcomes. SLEEP. 2025;48(4). doi: 10.1093/sleep/zsaf021 PMC11985401 39909735

[33] China Health and Retirement Longitudinal Study (CHARLS) [Internet]. 2019 Sep 13 [cited 2025 Oct 7]. Available from: http://charls.pku.edu.cn/pages/about/111/zh-cn.html

[34] von Elm E, Altman DG, Egger M, Pocock SJ, Gøtzsche PC, Vandenbroucke JP, et al. The Strengthening the Reporting of Observational Studies in Epidemiology (STROBE) statement: guidelines for reporting observational studies. J Clin Epidemiol. 2008;61(4):344–9. doi: 10.1016/j.jclinepi.2007.11.008 18313558

[35] Schokman A, Bin YS, Simonelli G, Pye J, Morris R, Sumathipala A, et al. Agreement between subjective and objective measures of sleep duration in a low-middle income country setting. Sleep Health. 2018;4(6):543–50. doi: 10.1016/j.sleh.2018.08.008 30442323

[36] Lauderdale DS, Knutson KL, Yan LL, Liu K, Rathouz PJ. Self-reported and measured sleep duration: how similar are they?. Epidemiology. 2008;19(6):838–45. doi: 10.1097/EDE.0b013e318187a7b0 18854708 PMC2785092

[37] Ding P, Li J, Chen H, Zhong C, Ye X, Shi H. Independent and joint effects of sleep duration and sleep quality on suboptimal self-rated health in medical students: A cross-sectional study. Front Public Health. 2022;10:957409. doi: 10.3389/fpubh.2022.957409 36276404 PMC9583520

[38] Ding R, Ding P, Tian L, Kuang X, Huang L, Shi H. Sleep duration trajectories and all-cause mortality among Chinese elderly: A community-based cohort study. BMC Public Health. 2023;23(1):1095. doi: 10.1186/s12889-023-15894-3 37344863 PMC10286431

[39] Rockwood K, Song X, MacKnight C, Bergman H, Hogan DB, McDowell I, et al. A global clinical measure of fitness and frailty in elderly people. CMAJ. 2005;173(5):489–95. doi: 10.1503/cmaj.050051 16129869 PMC1188185

[40] Liu X, Dai G, He Q, Ma H, Hu H. Frailty Index and Cardiovascular Disease among Middle-Aged and Older Chinese Adults: A Nationally Representative Cross-Sectional and Follow-Up Study. J Cardiovasc Dev Dis. 2022;9(7):228. doi: 10.3390/jcdd9070228 35877590 PMC9319589

[41] He D, Qiu Y, Yan M, Zhou T, Cheng Z, Li J, et al. Associations of metabolic heterogeneity of obesity with frailty progression: Results from two prospective cohorts. J Cachexia Sarcopenia Muscle. 2023;14(1):632–41. doi: 10.1002/jcsm.13169 36575595 PMC9891922

[42] Searle SD, Mitnitski A, Gahbauer EA, Gill TM, Rockwood K. A standard procedure for creating a frailty index. BMC Geriatr. 2008;8(1). doi: 10.1186/1471-2318-8-24 PMC2573877 18826625

[43] Song Y, Deng Y, Li J, Hao B, Cai Y, Chen J, et al. Associations of falls and severe falls with blood pressure and frailty among Chinese community-dwelling oldest olds: The Chinese Longitudinal Health and Longevity Study. Aging (Albany NY). 2021;13(12):16527–40. doi: 10.18632/aging.203174 34160365 PMC8266320

[44] Zhao Y, Hu Y, Smith JP, Strauss J, Yang G. Cohort profile: the China Health and Retirement Longitudinal Study (CHARLS). Int J Epidemiol. 2014;43(1):61–8. doi: 10.1093/ije/dys203 23243115 PMC3937970

[45] Nagin DS, Odgers CL. Group-based trajectory modeling in clinical research. Annu Rev Clin Psychol. 2010;6:109–38. doi: 10.1146/annurev.clinpsy.121208.131413 20192788

[46] Proust-Lima C, Philipps V, Liquet B. Estimation of Extended Mixed Models Using Latent Classes and Latent Processes: The R Package lcmm. J Stat Soft. 2017;78(2). doi: 10.18637/jss.v078.i02

[47] de Souza ÂMN, Fernandes DP de S, Castro IS, Gróla FG, Ribeiro AQ. Sleep quality and duration and frailty in older adults: a systematic review. Front Public Health. 2025;13:1539849. doi: 10.3389/fpubh.2025.1539849 40078770 PMC11898741

[48] Chung KW, Kim DH, Jung HJ, Arulkumar R, Chung HY, Yu BP. Chronic Inflammation as an Underlying Mechanism of Ageing and Ageing-Related Diseases. Subcell Biochem. 2023;103:31–44. doi: 10.1007/978-3-031-26576-1_3 37120463

[49] Balomenos V, Ntanasi E, Anastasiou CA, Charisis S, Velonakis G, Karavasilis E, et al. Association between sleep disturbances and frailty: evidence from a population-based study. Journal of the American Medical Directors Association. 2021;22(3):551-558.e1. doi: 10.1016/j.jamda.2020.08.012 32988763

[50] Chen T-Y, Lee S, Buxton OM. Multidimensional sleep health is associated with physical frailty in a national sample of Taiwanese community-dwelling older adults: Sex matters. Sleep Health. 2022;8(5):528–35. doi: 10.1016/j.sleh.2022.05.003 35794061

[51] Zhu Y, Fan J, Lv J, Guo Y, Pei P, Yang L, et al. Maintaining healthy sleep patterns and frailty transitions: a prospective Chinese study. BMC Med. 2022;20(1):354. doi: 10.1186/s12916-022-02557-0 36266610 PMC9585775

[52] Ensrud KE, Blackwell TL, Redline S, Ancoli-Israel S, Paudel ML, Cawthon PM, et al. Sleep disturbances and frailty status in older community-dwelling men. J Am Geriatr Soc. 2009;57(11):2085–93. doi: 10.1111/j.1532-5415.2009.02490.x 19793160 PMC3024909

[53] Sun M, Wang L, Wang X, Tong L, Fang J, Wang Y, et al. Interaction between sleep quality and dietary inflammation on frailty: NHANES 2005-2008. Food Funct. 2023;14(2):1003–10. doi: 10.1039/d2fo01832b 36546877

[54] He L, Yang J, Fang Y. Longitudinal analysis on inflammatory markers and frailty progression: based on the English longitudinal study of aging. Eur Geriatr Med. 2024;15(5):1323–30. doi: 10.1007/s41999-024-00998-9 38987423

[55] Piovezan RD, Abucham J, dos Santos RVT, Mello MT, Tufik S, Poyares D. The impact of sleep on age-related sarcopenia: Possible connections and clinical implications. Ageing Research Reviews. 2015;23:210–20. doi: 10.1016/j.arr.2015.07.003 26216211

[56] Irwin MR. Why Sleep Is Important for Health: A Psychoneuroimmunology Perspective. Annu Rev Psychol. 2015;66(1):143–72. doi: 10.1146/annurev-psych-010213-115205 25061767 PMC4961463

[57] Depner CM, Melanson EL, Eckel RH, Snell-Bergeon JK, Perreault L, Bergman BC, et al. Ad libitum weekend recovery sleep fails to prevent metabolic dysregulation during a repeating pattern of insufficient sleep and weekend recovery sleep. Current Biology. 2019;29(6):957-967.e4. doi: 10.1016/j.cub.2019.01.069 PMC12798825 30827911

[58] Havekes R, Abel T. The tired hippocampus: the molecular impact of sleep deprivation on hippocampal function. Curr Opin Neurobiol. 2017;44:13–9. doi: 10.1016/j.conb.2017.02.005 28242433 PMC5511071

[59] Niu S, Liu M, Lin Y, Gu P, Zhao L. The relationship between sleep disorders and frailty in stroke patients: the mediating role of self-efficacy. Front Psychiatry. 2025;16:1565412. doi: 10.3389/fpsyt.2025.1565412 40642415 PMC12240965

[60] Tan X, Chapman CD, Cedernaes J, Benedict C. Association between long sleep duration and increased risk of obesity and type 2 diabetes: A review of possible mechanisms. Sleep Med Rev. 2018;40:127–34. doi: 10.1016/j.smrv.2017.11.001 29233612

[61] Chinese Sleep Research Society. 2025 White Paper on National Health Sleep in China[Internet]. 2025 Mar 20 [cited 2025 Oct 7]. Available from: https://www.1848.cn/doc/44199.html

[62] Neikrug AB, Ancoli-Israel S. Sleep disorders in the older adult – a mini-review. Gerontology. 2009;56(2):181–9. doi: 10.1159/000236900 19738366 PMC2842167

[63] Hong Y, Xu W. Continuity and changes in grandchild care and the risk of depression for Chinese grandparents: new evidence from CHARLS. Front Public Health. 2023;11:1217998. doi: 10.3389/fpubh.2023.1217998 37601176 PMC10435994

[64] Li H, Ji Y, Chen T. The roles of different sources of social support on emotional well-being among Chinese elderly. PLoS One. 2014;9(3):e90051. doi: 10.1371/journal.pone.0090051 24594546 PMC3940715

[65] Rockwood K, Howlett SE. Age-related deficit accumulation and the diseases of ageing. Mech Ageing Dev. 2019;180:107–16. doi: 10.1016/j.mad.2019.04.005 31002924

[66] Nguyen QD, Moodie EM, Forget MF, Desmarais P, Keezer MR, Wolfson C. Health Heterogeneity in Older Adults: Exploration in the Canadian Longitudinal Study on Aging. J Am Geriatr Soc. 2021 Mar;69(3):678–87. doi: 10.1111/jgs.16919 33155270

