# Supplementary figures and images for: Association between nighttime sleep duration trajectories and frailty in middle-aged and older adults: A work-in-progress model based on a CHARLS cohort
Source: PLoS One. 2025 Dec 30;20(12):e0339843. doi: 10.1371/journal.pone.0339843 (PMC12753075; doi:10.1371/journal.pone.0339843)

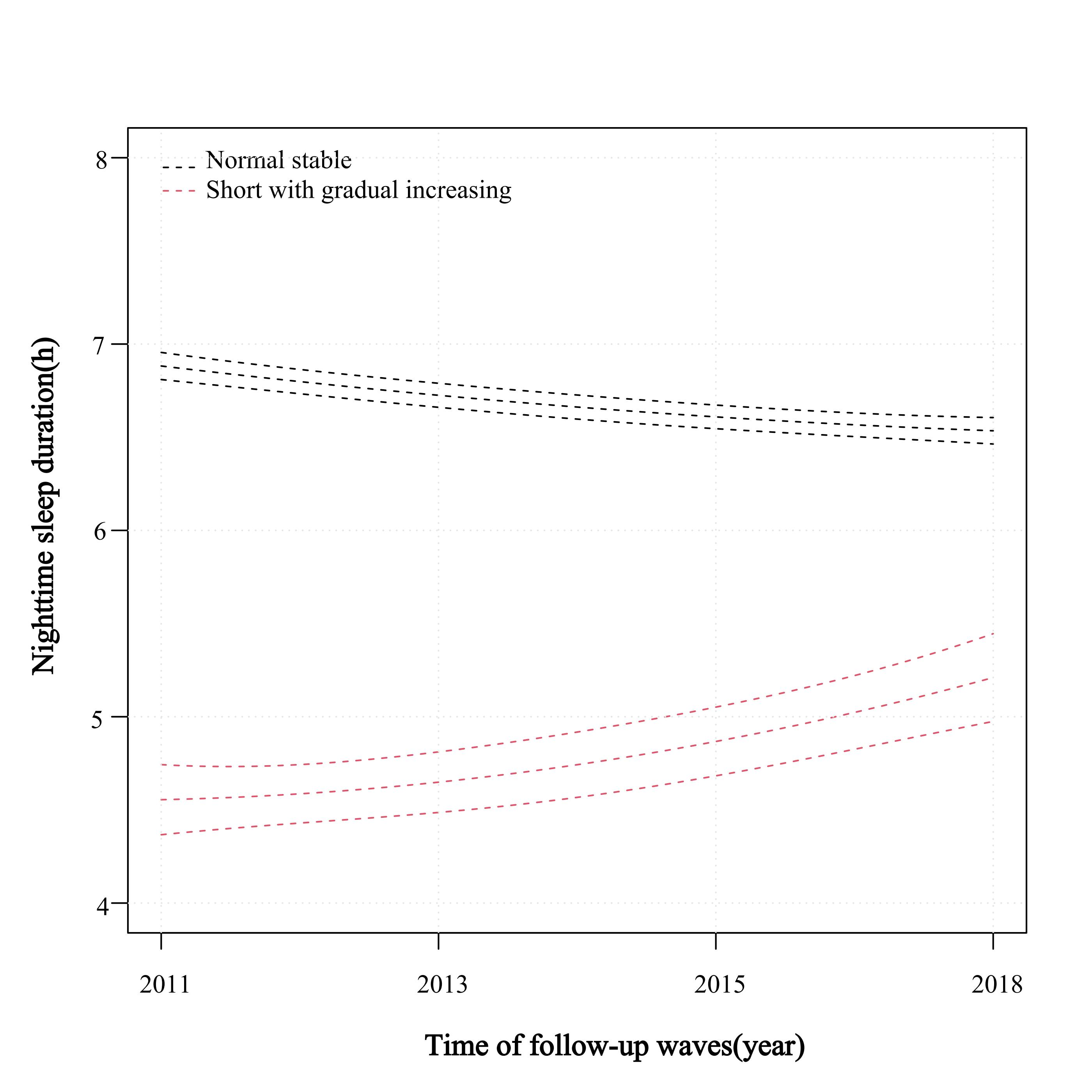

Supplement: S1 Fig — (TIFF) [file pone.0339843.s001.tiff]
