# Supplementary material for: Association between nighttime sleep duration trajectories and frailty in middle-aged and older adults: A work-in-progress model based on a CHARLS cohort
Source: PLoS One. 2025 Dec 30;20(12):e0339843. doi: 10.1371/journal.pone.0339843 (PMC12753075; doi:10.1371/journal.pone.0339843)
Supplement: S1 Table — (DOCX) [file pone.0339843.s002.docx]

**S1 Table Items constituting the Frailty Index and cut-off points.**

|  | **Items** | **Coding / Cut-off points** |
| --- | --- | --- |
| 1 | Self-reported physician diagnosed hypertension | Yes = 1, No = 0 |
| 2 | Self-reported physician diagnosed diabetes | Yes = 1, No = 0 |
| 3 | Self-reported physician diagnosed heart disease | Yes = 1, No = 0 |
| 4 | Self-reported physician diagnosed stroke | Yes = 1, No = 0 |
| 5 | Self-reported physician diagnosed cancer | Yes = 1, No = 0 |
| 6 | Self-reported physician diagnosed arthritis | Yes = 1, No = 0 |
| 7 | Self-reported physician diagnosed chronic lung disease | Yes = 1, No = 0 |
| 8 | Self-reported physician diagnosed asthma | Yes = 1, No = 0 |
| 9 | Self-reported physician diagnosed any emotional, nervous, or psychiatric problems | Yes = 1, No = 0 |
| 10 | Self-reported physician diagnosed memory-related disease | Yes = 1, No = 0 |
| 11 | Self-reported vision problems | Yes = 1, No = 0 |
| 12 | Self-reported hearing problems | Yes = 1, No = 0 |
| 13 | Self-reported general health status | 1= Poor, 0.75= fair; 0.5= good; 0.25= very good; 0= excellent |
| 14 | Difficulty with dressing | Yes = 1, No = 0 |
| 15 | Difficulty with bathing or showering | Yes = 1, No = 0 |
| 16 | Difficulty with eating | Yes = 1, No = 0 |
| 17 | Difficulty with getting in and out of bed | Yes = 1, No = 0 |
| 18 | Difficulty with using the toilet | Yes = 1, No = 0 |
| 19 | Difficulty with managing money | Yes = 1, No = 0 |
| 20 | Difficulty with taking medications | Yes = 1, No = 0 |
| 21 | Difficulty with shopping for groceries | Yes = 1, No = 0 |
| 22 | Difficulty with preparing meals | Yes = 1, No = 0 |
| 23 | Difficulty with doing housework | Yes = 1, No = 0 |
| 24 | Mobility: difficulty with walking 100 yards | Yes = 1, No = 0 |
| 25 | Mobility: difficulty with getting up from a chair after sitting for long periods | Yes = 1, No = 0 |
| 26 | Mobility: difficulty with climbing several flights of stairs without resting | Yes = 1, No = 0 |
| 27 | Mobility: difficulty with lifting or carrying weights over 10 pounds/jins | Yes = 1, No = 0 |
| 28 | Mobility: difficulty with picking up a coin from the table | Yes = 1, No = 0 |
| 29 | Mobility: difficulty with stooping, kneeling, or crouching | Yes = 1, No = 0 |
| 30 | Mobility: difficulty with reaching arms above shoulder level | Yes = 1, No = 0 |
| 31 | Depression: CESD-10 questionnaire | CESD-10 ≤ 10 =0 > 10 =1 |
| 32 | Cognition: (memory test score + orientation test score + serial 7's test + drawing test) / 21 | Continuous, ranging from 0 to 1 |
