# Supplementary material for: Association between nighttime sleep duration trajectories and frailty in middle-aged and older adults: A work-in-progress model based on a CHARLS cohort
Source: PLoS One. 2025 Dec 30;20(12):e0339843. doi: 10.1371/journal.pone.0339843 (PMC12753075; doi:10.1371/journal.pone.0339843)
Supplement: S2 Table — (DOCX) [file pone.0339843.s003.docx]

**S2 Table**  **Fit indices of latent class mixed model on nighttime sleep duration trajectories**

| **Model** | **Log likelihood** | **AIC** | **BIC** | **aBIC** | **Entropy** | **Class (%)** |
| --- | --- | --- | --- | --- | --- | --- |
| 1 | -62490.27 | 124994.50 | 125043.50 | 125021.30 | 1.00 | 1 |
| 2 | -62394.26 | 124810.50 | 124887.50 | 124852.50 | 0.62 | 88.30/11.70 |
| 3 | -62219.84 | 124469.70 | 124574.60 | 124527.00 | 0.75 | 10.92/87.50/1.57 |

Abbreviations: AIC, Akaike Information Criterion; BIC, Bayesian Information Criterion; aBIC, adjusted Bayesian Information Criterion.
