# Supplementary material for: Association between nighttime sleep duration trajectories and frailty in middle-aged and older adults: A work-in-progress model based on a CHARLS cohort
Source: PLoS One. 2025 Dec 30;20(12):e0339843. doi: 10.1371/journal.pone.0339843 (PMC12753075; doi:10.1371/journal.pone.0339843)
Supplement: S3 Table — (DOCX) [file pone.0339843.s004.docx]

**S3 Table Changes in nighttime sleep duration across trajectory groups from 2011 to 2018 (hours, Mean ± SD)**

| **Trajectory group** | **2011** | **2013** | **2015** | **2018** | **Mean change, hours (95% CI)** |
| --- | --- | --- | --- | --- | --- |
| **Normal stable trajectory group** | 6.88 ± 1.46 | 6.57 ± 1.52 | 6.70 ± 1.71 | 6.47 ± 1.85 | **-0.41 (-0.46, -0.37)** |
| **Short with gradual increasing trajectory** | 3.76 ± 1.19 | 3.87 ± 1.34 | 4.25 ± 1.66 | 4.88 ± 2.12 | **1.11 (0.97, 1.26)** |
