# Supplementary material for: Association between nighttime sleep duration trajectories and frailty in middle-aged and older adults: A work-in-progress model based on a CHARLS cohort
Source: PLoS One. 2025 Dec 30;20(12):e0339843. doi: 10.1371/journal.pone.0339843 (PMC12753075; doi:10.1371/journal.pone.0339843)
Supplement: S4 Table — (DOCX) [file pone.0339843.s005.docx]

**S4 Table Logistic regression models for frailty and nighttime sleep duration trajectories after excluding persons older than 80 years in the baseline survey**

|  | **Normal stable** | **Short with gradual increasing** |
| --- | --- | --- |
| Subjects, n | 7021 | 1019 |
| Frailty cases, n | 1290 | 307 |
| OR (95% CI) |  |  |
| Model 1^a^ | 0.632(0.543-0.736) | Reference |
| Model 2^b^ | 0.635(0.545-0.741) | Reference |
| Model 3^c^ | 0.711(0.608-0.831) | Reference |

^a^ Model 1 was adjusted for participants' demographic variables, including age, sex, residence, marital status, living arrangement, and educational level; ^b^ Model 2 was1 was additionally adjusted for health behaviors and socioeconomic factors (such as BMI, smoking, drinking, physical activity, household expenditure, and social engagement); ^c^ Model 3 was additionally adjusted for health-related factors (chronic pain) ; OR: odds ratio; CI: confidence interval.
